# Comparison of relapsing polychondritis patients with and without respiratory involvement based on chest computed tomography: a retrospective cohort study

**DOI:** 10.1186/s12890-022-01955-7

**Published:** 2022-06-08

**Authors:** Dong Wang, Lujia Guan, Xin Dong, Xiaofan Zhu, Zhaohui Tong

**Affiliations:** 1 Department of Respiratory and Critical Care Medicine, Beijing Institute of Respiratory Medicine and Beijing Chao-Yang Hospital, Capital Medical University, 8 Gongren Tiyuchang Nanlua, Chaoyang District, Beijing, 100020 China; 2 Department of Rheumatology and Autoimmune Diseases, Beijing Chao-Yang Hospital, Capital Medical University, Beijing, China; 3 Department of Medical Records Division, Beijing Chao-Yang Hospital, Capital Medical University, Beijing, China

**Keywords:** Relapsing polychondritis, Chest computed tomography, Respiratory involvement

## Abstract

**Background:**

Relapsing polychondritis (RP) patients with tracheal cartilage involvement are different from other patients. The objective of this study was to compare the clinical features and disease patterns between a respiratory involvement subgroup and a non-respiratory involvement subgroup according to chest computed tomography.

**Method:**

We performed a retrospective cohort study collecting RP patients hospitalized at the Beijing Chao-Yang Hospital between January 2012 and August 2021.

**Results:**

Respiratory involvement affected 59.7% of patients in our cohort. The incidence of costochondritis was more common in RP patients with respiratory involvement (*p* = 0.03); the incidence of inflammatory eye disease (*p* = 0.001) and auricular chondritis (*p* = 0.001) was less frequent in RP respiratory involvement patients.. Compared with the non-respiratory involvement subgroup the incidence of pulmonary infection marginally increased in the respiratory involvement subgroup (*p* = 0.06). Inflammatory indexes except for C-reactive protein to albumin ratio (CAR) were significantly higher in the respiratory involvement subgroup; analysis revealed no significant relationship between inflammatory indexes and pulmonary infection.

**Conclusion:**

RP patients with respiratory involvement had a greater incidence of costochondritis and pulmonary infectionand lesser incidence of inflammatory eye diseases and auricular chondritis compared to non-respiratory involvement. Increasing inflammatory indexes suggests that patients with respiratory involvement had a higher disease activity index of RP. The difference in probability of survival was insignificant between subgroups.

## Background

Relapsing polychondritis (RP) is a rare and systemic immune-mediated disease characterized by recurrent and progressive inflammation of the hyaline, elastic and fibrous cartilaginous structures, predominantly in the external ear, nose, and tracheobronchial tree [1]. Skin, cardiovascular system, nervous system, and blood system may  be affected. The first case of RP was reported by Jaksch-Wartenhorst in 1923, and Pearson et al. introduced the term "RP" and summarized the common clinical manifestations in 1960 [2]. A UK-based study found the incidence of RP between 1990 and 2012 was 0.71 per million populations per year with a standardized mortality ratio of 2.16 [3] and the prevalence of RP in the Department of Defense beneficiary population was 4.5 per million [4].

Respiratory involvement was present in up to 50% of RP patients [5, 6]. Symptoms such as cough, stridor, progressive dyspnea, and hoarseness were common in these patients many of whom were misdiagnosed as chronic bronchitis and refractory to conventional treatment leading to a diagnostic dilemma and poor prognosis. Chest Computed tomography (CT) could reveal the classic morphologic respiratory changes associated with RP. Our process in the present study was to retrospectively divide RP patients into two subgroups by chest CT findings, a subgroup of patients with respiratory involvement and a subgroup of patients with non-respiratory involvement, then describe and compare the clinical features and disease patterns of each subgroup.

## Methods

### Study design

We retrospectively recruited RP patients who were admitted to the Beijing Chao-Yang Hospital from January 2012 to August 2021. RP was diagnosed according to the diagnostic criteria proposed by McAdam et al. [7], Damiani and Levine [8] and Michet et al. [9] (Table 1). Patients were also included if they met the criteria for partial and limited RP [4, 10]. Respiratory manifestation and physical examinations, pulmonary function tests, CT imaging, bronchoscopy, and histological methods were used for airway. The study was conducted in accordance with the Declaration of Helsinki, and the protocol was approved by the Ethics Committee of the Chao-Yang Hospital (Project-ID 2021-12-1-1). Informed consent was waived because of the retrospective nature of this study.

**Table 1:** Three sets of diagnostic criteria for relapsing polychondritis

McAdam et al.	Bilateral auricular chondritis
	Nonerosive seronegative inflammatory arthritis
	Nasal chondritis
	Ocular inflammation
	Respiratory tract chondritis
	Audio-vestibular damage
	≥ Three symptoms
Damiani and Levine	Three symptoms of the McAdam's criteria
	One symptom of McAdam's criteria and histologic evidence of chondritis
	Chondritis at two or more separate anatomic locations with Response to corticosteroids, dapsone, or both
Michet et al	Two out of three symptoms: Auricular cartilage Inflammation, Nasal cartilage inflammation, Laryngotracheal cartilage inflammation
	One of the above and meeting two other signs: ocular inflammation, hearing loss, vestibular dysfunction, or seronegative inflammatory arthritis (any of these)

### Data acquisition

Data was obtained from the Electronic Medical Records (EMR). Patients younger than 18 years or without complete electronic case files were excluded. Patients with other immune-related diseases were also excluded. The clinical data was collected for all patients from medical records: patients’ profiles, clinical features, chest CT scans, blood tests, autoimmune series including rheumatoid factor (RF), antinuclear antibodies (ANA), anti-dsDNA antibodies, antineutrophil cytoplasmic antibodies (ANCA) and therapeutic interventions.

### CT image acquisition

Chest CT was performed using the same CT scanner (LightSpeed VCT, GE Healthcare, United States) with the following parameters: tube voltage, 120  kVp; 100–200 mAs tube current; 5.0-mm slice thickness with the 5.0-mm gaps. Image data was reconstructed by using the bone algorithm, with a slice thickness of 0.625 mm, and a slice number of about 400–460. Images were viewed at the standard soft-tissue window (level, − 40 HU; width, 350 HU) and lung window (level, − 700 HU; width, 1,500 HU).

### CT image analysis

The electronic scale from an open-source image-processing program (Image J Version 1.80, http://imagej.nih.gov/ij/) was used for measurement. We measured the thickness of the airway walls thickness from the trachea to the segmental bronchi. The total airway diameter and lumen diameter were measured at the level of the aortic arch, at the carina and 1 cm below the carina and segmental bronchial opening. Airway wall thickness was equal to (total airway diameter − lumen diameter)/2.

### Subgroup definition

Respiratory involvement in our study included airway wall thickening, calcification, and stenosis due to the lack of end-inspiratory and dynamic expiratory scans. Airway wall thickening was distinguished by the thickness of the wall in the involved airway segment being greater than 2 mm [11]. Airway calcification was recognized as nodular or linear hyperattenuating within the airway wall [12]. Airway stenosis (assessed by comparing the diameter of the involved segment with that of a corresponding uninvolved segment) was defined as at least a 25% reduction in the diameter of the lumen [11]. Diagnosis of pulmonary infection was made by a respirologist based on clinical manifestations, imaging findings, pathogen detection by culture methods, serologic examination results, and treatment effect observations.

### Statistical analysis

The descriptive analysis included the absolute and relative (percentage) frequencies for the categorical variables alongside the means (standard deviations, SD) and medians (interquartile ranges, IQR) for the quantitative parametric. Differences between the groups were computed using the Student's t-test or Fisher’s exact test for quantitative variables and the chi-square test for the qualitative variables. The Mann–Whitney or Kruskal–Wallis test was used for the non-normal variables. Kaplan–Meier survival analysis and Log-rank test were constructed to reveal survival analysis. Multiple imputations were performed on missing data. All analyses were performed using SPSS software version 21.0 and GraphPad Prism version 7.0. Two-tailed, *p* value < 0.05 was considered statistically significant.

## Results

**Fig. 1:**
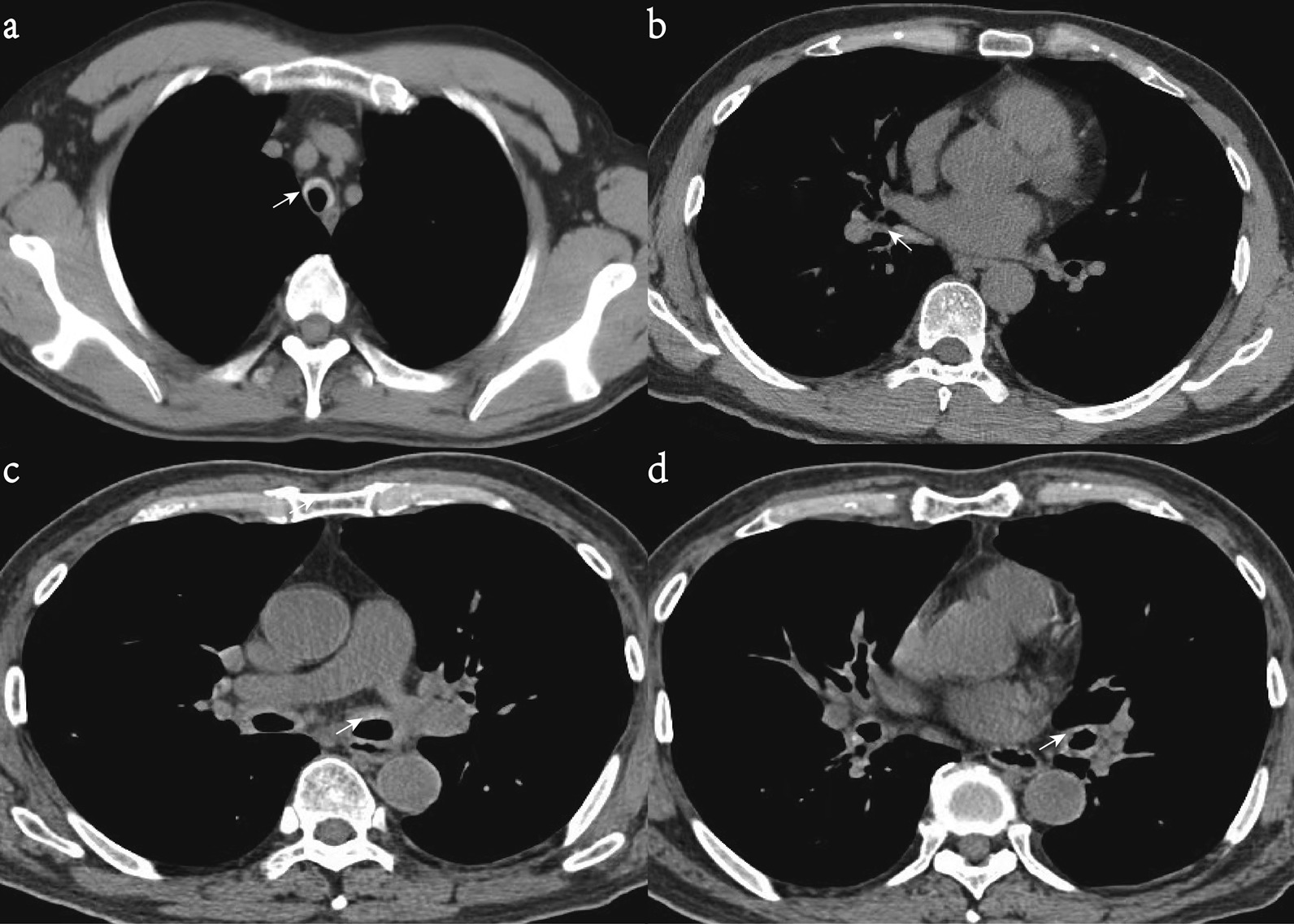
Axial CT slices showing thickening of the anterior and bilateral wall (arrow) of the upper trachea (**a**) in a 36-year-old woman with relapsing polychondritis. Axial CT slices showing thickening of the anterior and bilateral wall (arrow) of right inferior lobar bronchus (**b**), left main bronchus (**c**), left inferior lobar bronchus (**d**) in a 41-year-old man with relapsing polychondritis

**Fig. 2:**
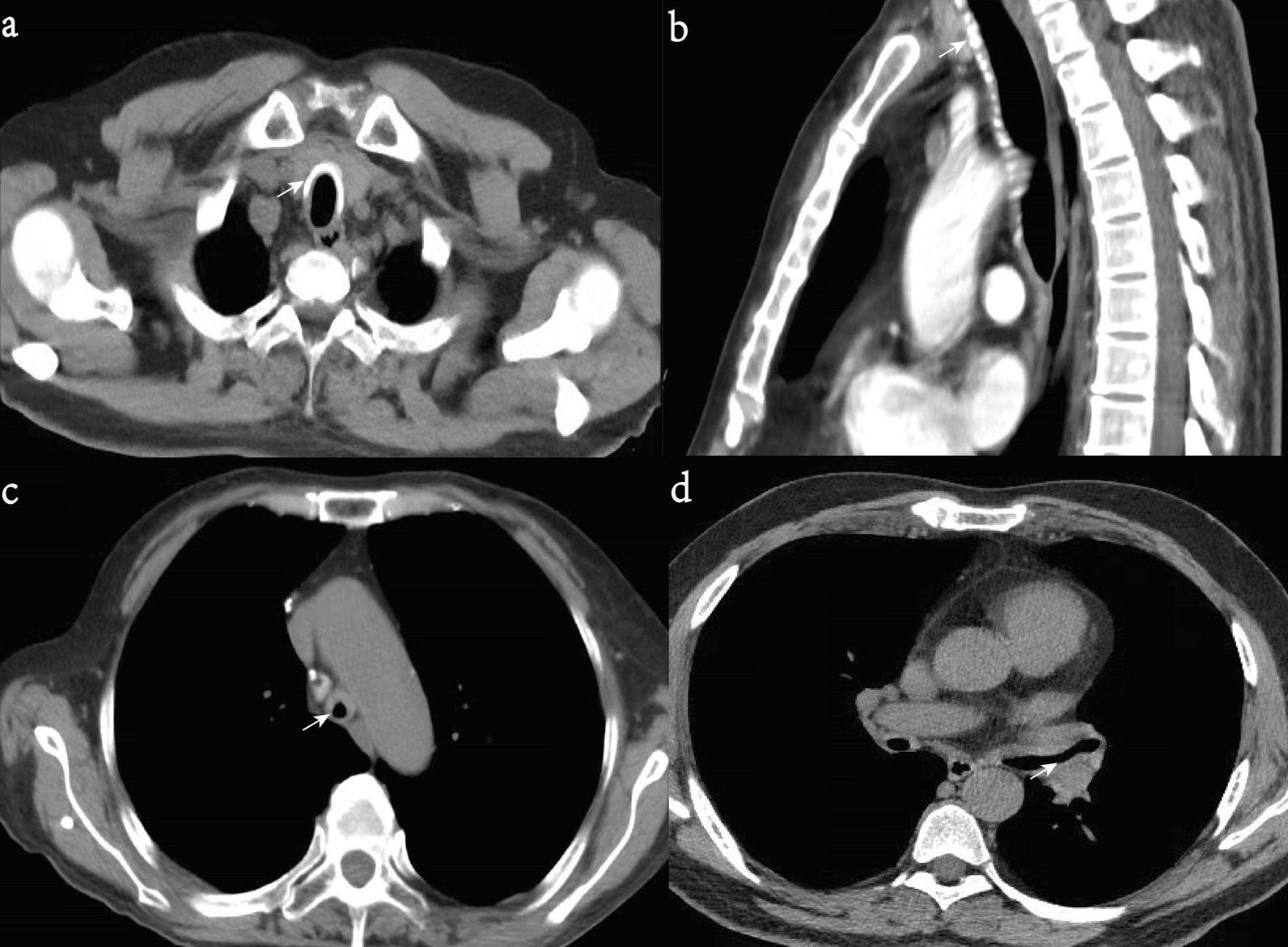
**a** Axial CT slices showing tracheal wall thickening contains calcific deposits (arrow). **b** Coronal CT reconstruction showing diffuse thickening of the anterior wall of the trachea, with calcifications (arrow) in a 47-year-old man with relapsing polychondritis. Axial CT slices showing airway stenosis (arrow) of the trachea (**c**) and left main bronchus (**d**) in a 56-year-old man with relapsing polychondritis

From January 2012 to August 2021, 75 patients with RP were screened in this study. 2 patients with positive ANCA against myeloperoxidase (MPO) diagnosed as Systemic vasculitis and 1 patient with positive ANCA against proteinase-3 (PR3) diagnosed as ANCA associated vasculitis were excluded. The diagnosis based on Damiani and Levine’s criteria was confirmed in 45 patients (62.5%),  Michet or Damiani criteria were fulfilled in 66 patients (91.6%), and 16 patients (22.2%) met all the three criteria. Although 6 (8.3%) patients did not fulfill any set of criteria, diagnosis was made based on  the improvement of auricular inflammation and respiratory symptoms after corticosteroid treatment. We reviewed previous chest CT images and found airway wall thickening sparing the posterior membranous wall was identified in 43 patients (59.7%) (shown in Fig. 1) and airway calcification sparing the posterior membranous wall was identified in 24 patients (33.3%) (shown in Fig. 2a and b); airway stenosis was identified in 10 patients (13.8%) (shown in Fig. 2c and d) while 29 (40.3%) patients did not show the above typical CT findings of RP. Altogether there were 43 (59.7%) and 29 (40.3%) patients in the respiratory involvement and non-respiratory involvement subgroups respectively. 64 patients received treatments with a base of prednisone of 0.5–1 mg/Kg/d; 18(25%) and  14(19.4%) of patients were being treated with methotrexate (MTX) and cyclophosphamide (CYC), respectively.

### Demographic characteristics

A total of 42 male and 30 female patients were included in our study and 29 of these 72 patients identified as smokers. The average age at the time of first symptoms was 54.03 ± 13.10 years and the average age at diagnosis was 55.20 ± 12.59 years. Delay from symptom onset to the diagnosis was 6 (2–24) months while follow-up visits after diagnosis averaged  4 (3–6) years. We did not find significant differences in other demographic features between  the two subgroups (Table 2).

**Table 2:** Comparison of patients with respiratory involvement and non- respiratory involvement

Variables	All patients (n = 72)	Non-airway involvement (n = 29)	Airway involvement (n = 43)	*p* value
Male	42(58.3%)	19(65.5%)	24(55.8%)	0.26
Smoke	29(40.3%)	11(37.9%)	18(41.9%)	0.73
Age at the time of first symptoms (years)	54.03 ± 13.10	56.36 ± 16.18	52.48 ± 10.71	0.39
The time of the first symptom to the diagnosis (months)	6(2–24)	4.5(2–15)	7(2–24)	0.52
Age at diagnosis(years)	55.20 ± 12.59	57.29 ± 15.44	53.81 ± 10.45	0.43
*Laboratory findings*				
IgG (mg/dl)	1139.82 ± 317.57	1062.2 ± 307.62	1187.87 ± 321.38	0.27
IgA (mg/dl)	272.5(182.5–370.25)	266(93.45–318)	277(190.5–405.5)	0.40
IgM (mg/dl)	78..35(61.45–130.25)	73.1(61.75–114.55)	82.8(60.2–132)	0.68
C3 (mg/dl)	109.72 ± 21.34	102.86 ± 18.31	113.97 ± 22.38	0.14
C4 (mg/dl)	28.8(22.45–34.18)	25.61 (22–30.65)	29.31(22.3–35.75)	0.26
Albumin (g/L)	36.31 ± 5.80	37.04 ± 7.36	35.85 ± 4.73	0.57
Total cholesterol (mmol/l)	4.23(3.85–5.09)	4.67(3.72–5.09)	4.12(3.83–5.11)	0.62
HDL-C (mmol/l)	1.1(0.8–1.59)	1.1(0.87–1.65)	1.02(0.77–1.54)	0.51
LDL-C (mmol/l)	2.75(2.48–3.32)	2.8(2.2–3.1)	2.72(2.53–3.77)	0.68
Triglyceride (mmol/l)	1.04(0.8–1.23)	1.09(0.85–1.34)	0.98(0.74–1.11)	0.08*
LDH (u/l)	190.5(158.75–216.25)	191(170–214.5)	190(140.5–229)	0.83
Creatinine (umol/l)	61.1(49.13–70.13)	69.8(50.5–79.35)	55.8(44.35–66.25)	0.02*
Uric acid (umol/l)	266.85 ± 89.99	320.15 ± 88.02	233.88 ± 75.66	0.01*
HbA1c (%)	5.95(5.52–7.34)	5.9(5.51–7.45)	6(5.55–7.57)	0.62
White blood cells (10^^9^)	8.06(6.54–10.05)	7.18 (5.64–10.66)	8.16 (7.04–9.57)	0.4
Neutrophils (10^^9^)	5.77(4.23–7.58)	5.61(3.05–7.07)	6.21(4.51–8)	0.23
Lymphocyte (10^^9^)	1.48(0.87–2.19)	1.85 (0.99–2.86)	1.32 (0.87–2.01)	0.29
Monocyte (10^^9^)	0.47 (0.41–0.66)	0.43 (0.38–0.72)	0.47 (0.44–0.68)	0.4
Hemoglobin (g/l)	124.4 ± 21.8	122.38 ± 28.68	123.95 ± 14.87	0.84
Platelet (10^^9^)	289.5(190.75–355.02)	245(175–311)	298(267.5–376.5)	0.15
CRP (mg/l)	2.31(0.61–8.19)	0.96(0.43–4.50)	3.96(1.74–9.78)	0.03*
ESR (mm/h)	25.5(13.75–44)	20 (13–28)	30(14–60.5)	0.04*
CAR	0.07(0.19–0.26)	0.23(0.14–0.11)	0.11(0.04–0.29)	0.04*
NLR	4.26(1.78–7.06)	1.80(1.47–6.08)	4.46(3.02–9.05)	0.03*
PLR	197.59(124.29–286.34)	132.43(76.10)-202.32	223.08(185.8–7-334.14)	0.01*
*Outcomes*				
Mortality	5(6.9%)	2(6.9%)	3(7%)	0.72

Data are presented as median (interquartile range), mean (standard deviation) or n (%)

HDL-C, high density lipoprotein cholesterol; LDL-C, low density lipoprotein cholesterol; LDH, lactate dehydrogenase; CRP, c-active protein; ESR, erythrocyte sedimentation rate; CAR, C reactive protein to albumin ratio; NLR, neutrophil to lymphocyte ratio; PLR, platelet to lymphocyte ratio

^*^p < 0.05

### Subgroup analyses of clinical characteristics and Laboratory findings

Respiratory involvement (n = 43) and auricular chondritis (n = 26) were the most frequent manifestations followed by ocular involvement consisting of scleritis and uveitis (n = 18), sensorineural hear loss (n = 18), costochondritis (n = 14), nasal chondritis (n = 11). Patients in the respiratory involvement subgroup had a significantly higher occurrence of costochondritis (*p* = 0.03) compared with the  occurrence of auricular chondritis (*p* = 0.001) or inflammatory eye disease (*p* = 0.001). Laryngeal involvement in 8 patients, inflammatory arthritis in 6 patients and cutaneous manifestations in 4 patients were other common symptoms although there was no significant difference in these  clinical manifestations between the two groups. Although not statistically significant, there was a clear trend developing toward a higher frequency of pulmonary infection in respiratory involvement subgroups (*p* = 0.06) (Table 3). Cardiac involvement in the form of myocardial infarction or ventricular tachycardia was seen in 4 patients. One patient developed cytopenia after treatment with glucocorticoids and immunosuppressants; a follow-up bone marrow biopsy discovered bone marrow suppression.

**Table 3:** Differences in Clinical features among the 2 subgroups of patients with RP

Variables	All patients (n = 72)	Non-airway involvement (n = 29)	Airway involvement (n = 43)	*p* value
*Clinical features, n (%)*				
Auricular chondritis	26 (36.1%)	19 (65.5%)	7 (16.2%)	0.001*
Fever	26 (36.1%)	11 (37.9%)	15 (34.9%)	0.79
Inflammatory eye disease	18 (25%)	13 (44.8%)	5 (11.6%)	0.001*
Sensorineural hear loss	18 (25%)	10 (34.5%)	8 (18.6%)	0.13
Costochondritis	14 (19.4%)	2 (6.9%)	12 (27.9%)	0.03*
Pulmonary infection	14 (19.4%)	3 (10.3%)	11 (25.6%)	0.06
Nasal chondritis	11 (15.3%)	4 (13.8%)	7 (16.3%)	0.77
Laryngeal involvement	8 (11.1%)	2 (6.9%)	6 (14%)	0.35
Inflammatory arthritis	6 (8.3%)	2 (6.8%)	4 (9.3%)	0.72
Cutaneous manifestations	4 (5.6%)	2 (6.9%)	2 (4.7%)	0.68
Myocardial infarction	4 (5.6%)	2 (6.9%)	2 (4.7%)	0.65

Data are presented as n (%); patients may have more than one symptom at the same time

*p < 0.05

### Comparisons of laboratory findings and subgroup analysis

The respiratory involvement subgroup had higher C-reactive protein (CRP) and erythrocyte sedimentation rate (ESR) than the non-respiratory involvement subgroup (*p* = 0.03, *p* = 0.04). Creatinine and Uric acid in the non-respiratory involvement subgroup were significantly higher than in the respiratory involvement subgroup (*p* = 0.02, *p* = 0.01). Novel inflammatory markers associated with RP disease activity index: neutrophil to lymphocyte ratio (NLR), platelet to lymphocyte ratio (PLR) were higher in the respiratory involvement subgroup (*p* = 0.03, *p* = 0.01), but CAR was lower (*p* = 0.04). We conducted a subgroup analysis and found no statistical difference in inflammatory indicators between respiratory involvement patients with pulmonary infection and respiratory involvement patients without pulmonary infection (Table 4).

**Table 4:** Subgroup analysis of respiratory involvement patients with pulmonary infection or without pulmonary infection

	Without pulmonary infection (n = 32)	With pulmonary infection (n = 11)	*p* value
CRP	4.35 (2–8.4)	3.49 (0.7–10.7)	0.81
ESR	37.5 (25–58.3)	24 (13–62)	0.46
CAR	0.11 (0.05–0.31)	0.09 (0.02–0.29)	0.38
NLR	4.11 (2.51–10.04)	5.06 (3.91–8.23)	0.55
PLR	243.8 (185.45–453.12)	209.85 (181.31–288.53)	0.91

Data are presented as median (IQR)

CRP, c-active protein; ESR, erythrocyte sedimentation rate; CAR, C reactive protein to albumin ratio; NLR, neutrophil to lymphocyte ratio; PLR, platelet to lymphocyte ratio

Pulmonary function tests (PFTs) were performed in 22 patients with respiratory involvement and showed respiratory obstruction in all patients, obviously reducing in forced expiratory volume in 1 s (FEV1): 1.21 ± 0.54L, forced expiratory volume in 1 s/forced vital capacity (FEV1/FVC): 42.11 ± 14.84%, residual volume (RV), total lung capacity (TLC), residual volume /total lung capacity (RV/TLC) were usually normal. Positive ANA were found in 8 patients in a titre of 1: 320. RF was positive in 4 patients, and ANCA were positive in 4 patients but no particular specificities were found.

### Survivals

**Fig. 3:**
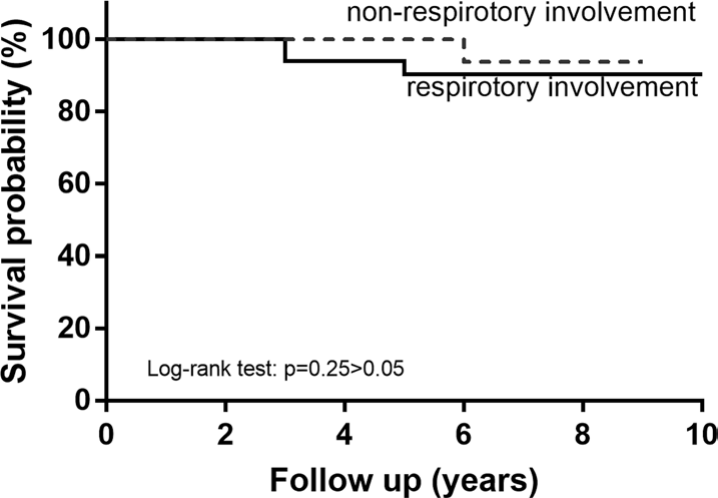
Kaplan–Meier survival curve and Log-rank test of respiratory involvement and non-respiratory involvement RP patients. *p < 0.05

After a follow-up period of 6 (3–8) years since the first symptoms occurred, 6.9% of the patients (n = 5) died (Table 2). K–M curve and Log-rank test also showed the probability of survival was not statistically different between patients with and without respiratory involvement (shown in Fig. 3).

## Discussion

In our study we did not find significant differences in the age of disease onset, disease duration, age at diagnosis, or the male-to-female ratio between the two groups which was consistent with previous studies [13–15]. RP patients with respiratory involvement in our cohort demonstrated elevation of airway involvement compared with that in other studies [16, 17]. The incidence of inflammatory eye disease and auricular chondritis was significantly higher in the non-respiratory involvement subgroup than in the respiratory involvement subgroup. The respiratory involvement subgroup trended towards significantly increased the likelihood of pulmonary infection. CRP, ESR, and novel inflammatory markers associated with RP activity index (NLR, PLR) were higher in the respiratory involvement subgroup but CAR was lower. Probability of survival was not statistically different between subgroups. This is the first study analyzing and comparing the clinical features and disease patterns in RP patients with and without respiratory involvement based on chest CT findings.

RP is a rare autoimmune disease characterized by recurrent inflammation and destruction of cartilage [18]. The cause of RP is uncertain but regarded as autoimmune-mediated [19]. Although anti-Collagen Type II antibodies, matrilin-1, and cartilage oligomeric matrix proteins could be found in the targeted cartilage, the above antibodies are not specific and not used in clinical practice [19–23]. There is no validated classification for RP, the diagnosis depends on a combination of clinical representation, imaging, and biopsy of involved cartilaginous tissues. Ernst [17] investigated 145 RP patients and found that 21% of patients had respiratory involvement with respiratory symptom usually the first symptom in 54% of these patients. Dion [24] performed a retrospective study of 142 RP patients in France using  a cluster analysis to identify three separate phenotypes: hematological phenotype, respiratory phenotype, and mild phenotype; this analysis discovered that 22% of RP patients exhibited respiratory manifestations and that patients in the respiratory phenotype frequently received intensive treatments and suffered more infections which has been considered a significant cause of mortality in previous studies. Recently, a study [25] involving 239 RP patients found 19.7% patients in the respiratory involvement without auricular involvement subgroup and 29.3% of patients in both respiratory and auricular involvement subgroups. The above studies defined respiratory involvement by the patterns of clinical manifestations. Meanwhile, a good agreement was found between clinical and radiographic data for the diagnosis and assessment of disease activity in RP patients [26]. Another review [11] suggested that chest CT scans, particularly with end-inspiratory and dynamic expiratory scans should be routinely performed at diagnosis and during the evolution of the disease. Malacia or air trapping may be the a unique CT abnormality [16, 27] that can be more easily detected during the dynamic expiration scan. In our study, 43 patients were found with thickening of anterior and lateral respiratory walls, which is considered to be the early stage of the disease [23]; increased attenuation of the cartilages sparing the posterior membranous wall was identified in 24 patients and respiratory stenosis was recognized in 10 patients.

Consistent with studies in the French and Japanese patients [24, 25], our current study demonstrated obvious differences between two subgroups in terms of clinical characteristics: The respiratory involvement subgroup was characterized by laryngotracheal chondritis and costochondritis while the non-respiratory involvement subgroup was distinguished by inflammatory eye disease and auricular chondritis. We also found five patients with auricular chondritis or inflammatory eye diseases that were assigned to the respiratory involvement subgroup because their chest CT discovered airway thickening, calcification and stenosis. We did not complete a further subgroup analysis on account of the small quantity; those patients need to be analyzed independently as well as in the overlapping subgroup [25]. In contrast to other studies [4, 7, 28–30], involvements of skin (5.6%: 13–46%) and cardiovascular involvements (5.6%: 2–31%) were less common. Chang-Miller et al. [31] reported renal involvement in 29 of 129 patients. Although renal involvement was not presented in our study, uric acid and creatinine levels were higher in the non-respiratory involvement subgroup as signs of kidney damage.

Although RP is considered to be an immune system disease, no significant abnormalities have been found in either cellular or humoral immunity. CRP and ESR are markers of the severity of common rheumatic diseases, higher CRP and ESR concentrations in the respiratory involvement subgroup suggest more inflammation and disease activity. Novel inflammatory markers were associated with activity in many rheumatic diseases [32, 33]. Cao [34] indicated that CAR, NLR and PLR levels were significantly higher in RP patients than in healthy controls and were positively correlated with relapsing polychondritis disease activity index (RPDAI). We found that NLR and PLR were higher in the respiratory involvement subgroup but CAR was lower compared to the non-respiratory involvement subgroup. Subgroup analysis of patients with respiratory involvement found that there was no statistical difference between pulmonary infection and inflammation indexes, suggesting those patients had a high level of disease activity.

### Limitation of the study

Our study had some limitations. Firstly, it was a single-center, retrospective study, with a small sample size. Secondly, air trapping or mosaic sign usually occurred in the early stages of RP. While RP was diagnosed without dynamic expiration, air trapping or mosaic sign cannot be detected in time and may result in a delay of diagnosis.

## Conclusions

According to chest CT finding, 59.7% of patients had respiratory involvement in our cohort. RP patients with respiratory involvement were characterized by a higher rate of costochondritis and pulmonary infection, fewer inflammatory eye diseases and auricular chondritis compared to non-respiratory involvement. Elevated inflammatory indexes suggests that patients with respiratory involvement had a higher disease activity index of RP. The probability of survival was not significantly different between the two subgroups.

## Data Availability

Raw data is available from the corresponding author on reasonable request.

## Abbreviations

RP: Relapsing polychondritis
CAR: C-reactive protein to albumin ratio
CT: Computed tomography
EMR: Electronic Medical Records
RF: Rheumatoid factor
ANA: Antinuclear antibodies
ANCA: Antineutrophil cytoplasmic antibody
SD: Standard deviations
IQR: Interquartile ranges
MPO: Myeloperoxidase
PR3: Proteinase-3
MTX: Methotrexate
CYC: Cyclophosphamide
CRP: C-reactive protein
ESR: Erythrocyte sedimentation rate
NLR: Neutrophil to lymphocyte ratio
PLR: Platelet to lymphocyte ratio
PFTs: Pulmonary function tests
FEV1: Forced expiratory volume in 1s
FVC: Forced vital capacity
RV: Residual volume
TLC: Total lung capacity
RPDAI: Relapsing polychondritis disease activity index
